# Draft Genome Sequences of Five Clinical Methicillin-Resistant *Staphylococcus aureus* Isolates and a Methicillin-Resistant *Staphylococcus epidermidis* Isolate

**DOI:** 10.1128/genomeA.00836-15

**Published:** 2015-07-30

**Authors:** Rajesh Kumar Biswas, Marleen M. Kock, Toyin Adelowotan, Wilhelmina Strasheim, Tanweer Goolam Mahomed, Adeola Salawu, Marthie M. Ehlers

**Affiliations:** a Department of Medical Microbiology, University of Pretoria, Pretoria, South Africa; b Genomics Research Institute, University of Pretoria, Pretoria, South Africa; c Department of Medical Microbiology, Tshwane Academic Division, National Health Laboratory Service, Pretoria, South Africa

## Abstract

We report the complete draft genome sequences of five individually isolated strains of methicillin-resistant *Staphylococcus aureus* (MRSA) and a *Staphylococcus epidermidis* strain. These clinically important isolates have staphylococcal cassette chromosome *mec* type A, while Panton-Valentine leukocidin (PVL) toxin coding genes were present in MRSA isolates only.

## GENOME ANNOUNCEMENT

*Staphylococcus* species can cause various forms of infection (1). *S. aureus* is an important nosocomial and community associated pathogen. *S. epidermidis* causes nosocomial infections by forming biofilms on invasive medical devices (2–5). *S. aureus* produces several exotoxins, including β-pore-forming toxins such as Panton-Valentine leukocidin (PVL) (1). The ability to produce toxins are mostly associated with increased virulence (6). The staphylococci can colonize people for years, in the process spreading from person to person and evolving genetically to become unique (7).

We have sequenced and assembled the complete genomes of clinically isolated methicillin-resistant *S. aureus* (MRSA) and *S. epidermidis* (MRSE), in order to better understand the genetic components of antibiotic (methicillin) resistance and various associated virulence factors*.* Clinical specimens were cultured on blood agar, and the genomic DNA (gDNA) was isolated using the Zymo Research Fungal/Bacterial mini prep kit. The integrity of the gDNA was analyzed by spectrophotometry, as well as on agarose gel electrophoresis. The gDNA samples were subjected to whole-genome sequencing using the Illumina MiSeq version 3 (2 × 300 bp) kit on the Illumina sequencing platform (outsourced to Inqaba Biotech, http://www.inqababiotec.co.za). This resulted in approximately 5 billion short read sequences in pairs of ~300 bp, corresponding to different isolates. The generated short reads were processed (QC), assembled and analyzed using CLC Genomics workbench version 7.0 and other open-source software programs on windows or Bio-Linux platforms (8). The *de novo* assembly of the processed reads generated several contigs. Newly assembled contigs were validated by matching against reference genomes by BLAST at NCBI or locally. Contigs were further mapped and aligned to the reference genome sequence of *S. aureus* MRSA (NCBI reference sequence NC_002952) or the *S. epidermidis* reference genome (NCBI reference sequence NC_004461) using CONTIGuator2 (9) to obtain a scaffolded pseudocontig molecule for NCBI submission.

The assembled genomes were approximately 2.7 Mb in length, with an average G+C content of ~33.0%. The average coverage depth of more than 30× was maintained at most of the regions. The genomes were annotated using the NCBI microbial genome annotation pipeline. The genomes were annotated with 2,119 to 2,675 coding DNA sequences (CDSs), among which were 31 to 56 tRNA-coding genes and 3 to 5 rRNA-coding genes, while 41 to 121 genes were annotated as pseudogenes. Initial comparative analyses with the *S. aureus* MRSA and *S. epidermidis* reference genomes highlighted various phage integration sites, genomic islands, indels, and frameshifted genes. The detailed genomic data analysis from these *de novo* genome assemblies is under way to obtain a finer resolution of methicillin antibiotic resistance in MRSA and *S. epidermidis* at the nucleotide level.

### Nucleotide sequence accession numbers.

This whole-genome shotgun project has been deposited at NCBI GenBank under the accession numbers CP010940, CP010941, CP010942, CP010952, CP010943, and CP010944 under Bioproject PRJNA274731. The versions (first) described in this paper are the latest GenBank versions.
